# Adverse Effects Associated with Proton Pump Inhibitor Use

**DOI:** 10.7759/cureus.12759

**Published:** 2021-01-18

**Authors:** Marcel Yibirin, Diana De Oliveira, Roberto Valera, Andrea E Plitt, Sophia Lutgen

**Affiliations:** 1 Department of Infectious Diseases, Infection Control, and Employee Health, University of Texas MD Anderson Cancer Center, Houston, USA; 2 Department of Research, Foundation for Clinic, Public Health, and Epidemiological Research of Venezuela (FISPEVEN), Caracas, VEN; 3 Department of General Surgery, Cleveland Clinic Florida, Weston, USA; 4 Critical Care, Dr. Ignacio Pirovano Hospital, Buenos Aires, ARG; 5 Internal Medicine, Dr Juan A. Fernández Hospital, Buenos Aires, ARG

**Keywords:** proton pump inhibitors, adverse effects

## Abstract

Proton pump inhibitors (PPIs) marked a before and after in the management of gastric acid‐related disorders since their introduction to the market in 1989. Due to a novel, highly effective mechanism of action blocking the last converging step of gastric acid secretion by parietal cells and very few and mostly tolerable side effects, these drugs quickly displaced other pharmacological compounds such as H2 antagonists as the first treatment choice for peptic ulcer disease, gastroesophageal ulcers, Zollinger-Ellison syndrome, nonsteroidal anti-inflammatory drug-associated ulcers, and eradication of Helicobacter pylori, leading to an exponential increase in their prescription up to now. However, widespread PPI use has led to emerging evidence of long-term adverse effects not described previously, including increased risk of kidney, liver, and cardiovascular disease, dementia, enteroendocrine tumors of the gastrointestinal tract, susceptibility to respiratory and gastrointestinal infections, and impaired absorption of nutrients. Although the evidence published thus far has not established strong correlations, it has been relevant enough to raise new questions about PPIs’ safety profile and reconsideration of their clinical indications. Hence, the aim of this review is to evaluate the association between PPI use and the risk of serious adverse effects given increasing concerns about the overuse of PPIs in the general population.

## Introduction and background

**Figure 1:**
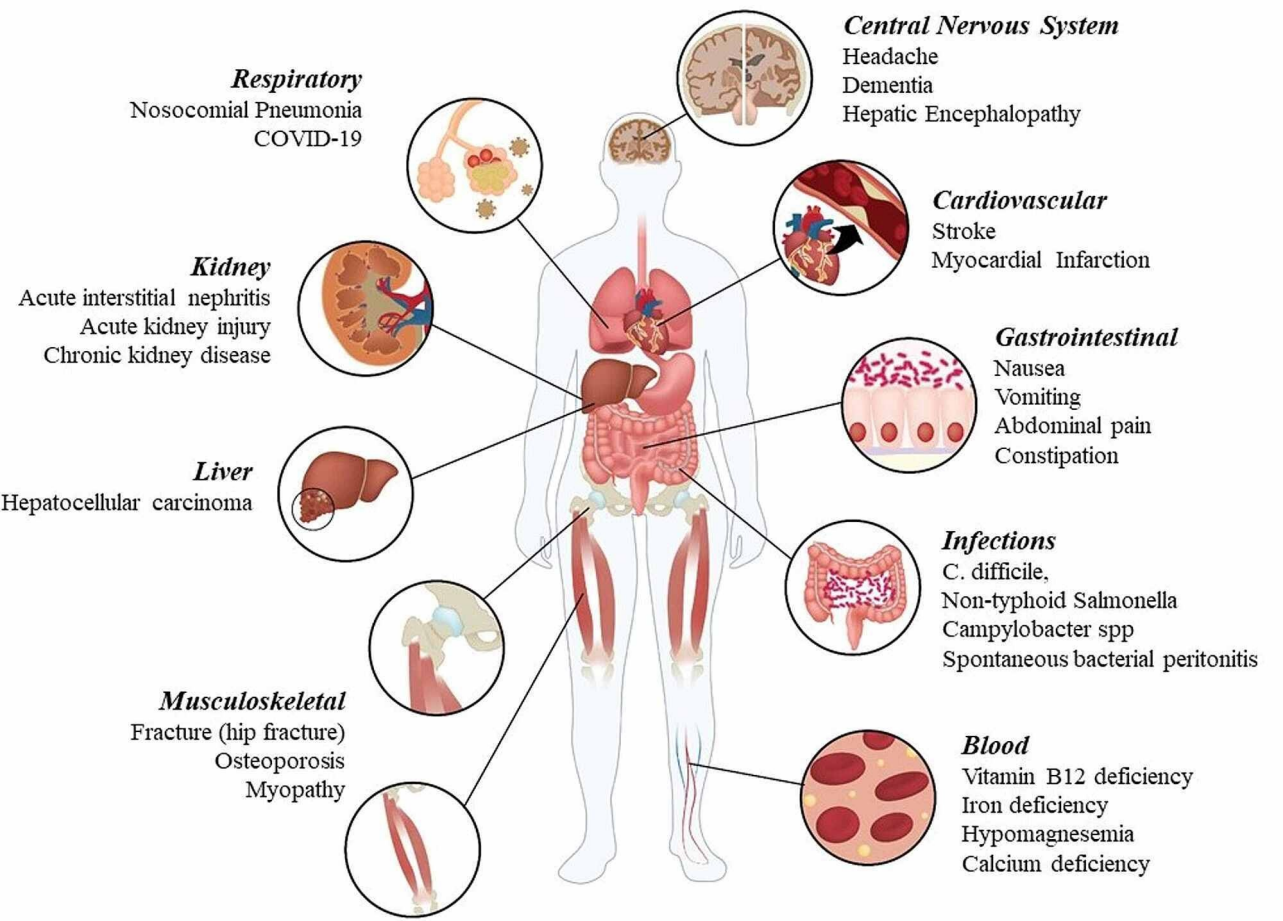
Side effects associated with the use of proton pump inhibitors

Proton pump inhibitors (PPIs) are widely used irreversible inhibitors of H+/K+ adenosine triphosphatase (ATPase), the final step of gastric acid secretion by parietal cells in the stomach. Over the past few decades, the use of these drugs has increased in many countries due to the expansion of their role as drugs of choice in the treatment of gastric acid‐related disorders such as peptic ulcer disease, gastroesophageal ulcers, Zollinger-Ellison syndrome, nonsteroidal anti-inflammatory drug-associated ulcers, and eradication of Helicobacter pylori. In the United States, the use of PPIs doubled from 3.9% in 1999 to 7.8% in 2012. However, numerous studies have demonstrated overprescription of PPIs [1]. In general, PPIs are believed to have few adverse effects, as they are generally well tolerated. Patients have experienced few minor side effects of short-term PPI use, such as headache, rash, dizziness, and gastrointestinal symptoms including nausea, abdominal pain, flatulence, constipation, and diarrhea. In general, physicians are not concerned about serious side effects of PPIs at approved dosing during a brief treatment time of about two weeks, but as the use of these drugs increases, reports of their side effects are increasing, particularly with long‐term use [2]. In recent studies, researchers advised that PPIs should be used for the shortest time period at the smallest effective dose [3], as infections, impaired absorption of nutrients, dementia, kidney disease, and hypergastrinemia-related side effects are emerging as possible consequences of long-term use [2]. Therefore, the aim of this review is to describe the association between PPI use and the risk of serious adverse effects given the increasing concerns about the overuse of PPIs in the general population (Figure 1).

## Review

Kidney disease

Since 1992, case reports have linked PPI use with acute kidney injury [1], and recently, two studies connected PPI use with an excessive risk of chronic kidney disease (CKD), which was not explained solely by the risk of acute kidney injury, with evidence that patients who used PPIs for longer durations had higher risk of CKD [4]. Apparently, patients with established diagnoses of CKD may progress rather quickly on PPI therapy [5-7]. The main mechanism leading to renal pathology due to PPI use could be acute interstitial nephritis. More than half of the patients who suffered PPI-induced acute interstitial nephritis [7] did not fully recover, suggesting that PPI-induced CKD is due to progression of acute interstitial nephritis with inflammatory interstitial infiltrates and edema to chronic interstitial scarring and tubular atrophy. Taken together, these findings represent good evidence that PPIs cause acute interstitial nephritis and some evidence that they also increase the risk of CKD. Initially, physicians considered PPIs to also inhibit other than gastric proton pumps, such as the ones in the renal tubule, but definitive evidence of this in a clinical setting is lacking [8-9].

Infections

Gastrointestinal Infections

PPI use has been linked with increased risk of both incidental and recurrent Clostridium difficile infections [10-13]. Acid secretion by parietal cells is an important immunological barrier in the gastrointestinal tract, which is why hypochlorhydria generated by the inhibition of gastric acid secretion increases the risk of bacterial colonization and altered intestinal flora and susceptibility to enteric infections [2]. Studies have demonstrated that intense suppression of gastric acid secreation is associated with increased risk of Clostridium difficile infection. Authors reported that more than two-thirds of inpatient acid-suppressive prescriptions are not strictly indicated and the number of Clostridium difficile infection cases in the United States is in the tens of thousands per year [14]. Authors have reported other enteric infections, such as non-typhoid Salmonella and Campylobacter infections [1-2,15], the latter of which has been racing [2].

Respiratory Infections

Pneumonia has been widely associated with PPI use, especially over the short term (usually fewer than 30-90 days). However, a recent meta-analysis demonstrated that this association may be overestimated [16]. The most likely explanation for the increased risk of respiratory infections with PPI use is that PPI-induced hypochlorhydria increases microaspiration of gastric contents, which increases lung colonization and the subsequent incidence of pneumonia [2]. PPI use also maybe associated with an increased risk of coronavirus disease 2019 (COVID-19), as authors have reported that patients using these drugs had higher odds of testing positive for COVID-19 than did patients not taking PPIs [17]. Coronaviruses are easily destroyed by an acidic gastric pH, although the impact of acid suppression is unclear. Previously reported data suggested that the infectivity of severe acute respiratory syndrome coronavirus 1 was reduced in individuals with a gastric pH of up to 3 [17]. Furthermore, researchers have shown coronaviruses to survive in individuals with more basic gastric pH levels, including those in the range created by the use of drugs like omeprazole and esomeprazole [17]. Current and past PPI use were associated with poor outcomes of COVID-19. However, this PPI use did not increase susceptibility to severe acute respiratory syndrome coronavirus 2 infection [16]. Receptors for COVID-19 (ACE-2) are found throughout the gastrointestinal tract, making plausible the hypothesis that PPI users may be more vulnerable than nonusers to the effect of high viral loads [16].

Gastrointestinal malignancies

Because PPIs decrease gastric acid secretion, compensatory raising of gastrin levels in patients has a proliferative effect on the growth of enterochromaffin-like cells, explaining the association of PPIs with development of neuroendocrine tumors and carcinomas of the gastrointestinal tract [18-20]. Another related mechanism is that PPIs facilitate gastric pan-colonization by Helicobacter pylori due to a decrease in the normal stomach acidic environment [1]. Numerous mechanistic studies suggested that hypoacidity and hypergastrinemia increase the risk of gastric cancer in the corpus/fundus, which was supported by epidemiological studies.

Liver disease

PPI use has been linked with increased risk of cirrhosis-related complications such as hepatic encephalopathy, spontaneous bacterial peritonitis, and liver cancer [21-24]. These effects appear to be related to chronic PPI use, as patients who underwent more than one year of follow-up after initiating treatment with PPIs had twice the risk of hepatocellular carcinoma than did those with no more than one year of follow-up [3]. The mechanism of liver injury associated with PPI use, is not completely understood, although investigators observed that H+/K+ ATPase inhibition leads to intestinal bacterial overgrowth and an altered intestinal microbial composition [25], which may lead to increased portal venous concentrations of several potentially harmful substances, including secondary bile acids [26]. PPIs are metabolized in the liver; therefore, patients with liver disease may be at risk for increased hepatotoxicity, which can lead to hypergastrinemia-induced carcinogenic effects, especially on liver cells [27-28]. Finally, the authors reported that after exposure to PPIs, cultured human liver cells exhibited gene expression similar to well-known carcinogens in the liver [29].

Fracture risk

Increased fracture risk due to PPI intake is a controversial topic [30]. Retrospective studies have suggested the existence of a dose-dependent relationship between PPIs and decreased bone mineral density, leading to an increase in fracture risk, especially hip fractures. The risk appears to be higher in patients with a risk factor for osteoporosis, such as renal dysfunction. Routine prophylaxis for osteoporosis is suggested for PPI users to preventosteoporotic fractures [30-32]. However, more recent prospective studies showed no significant changes in bone mineral density or fracture risk in PPI users over the short to medium term [33,34]. Proposed mechanisms that link long-term PPI-based therapy with decreased bone mineral density include hypochlorhydria-associated malabsorption of calcium (absorption of which is indispensable to maintaining bone microstructure), gastrin-induced parathyroid hyperplasia, and inhibition of bone resorption by blocking local H+/K+ ATPase [35-37].

Dementia

Overall, data on the association between PPI use and dementia risk are conflicting. Physicians have yet to come to a consensus on the role of PPIs and the associated risk of dementia. Even with uncertainty about the mechanism, most cases of brain dysfunction in PPI users are reported to be associated with chronic administration of PPIs [38]. Some PPIs, such as lansoprazole, esomeprazole, and pantoprazole, have been linked with neurological side effects, such as headaches and dizziness/vertigo. Less common reported side effects involving the central nervous system include depression, diplopia, disturbed sleep, drowsiness, insomnia, nervousness, tremor, sensory and perceptual abnormalities (e.g., hallucinations), and delirium [39]. Although the mechanisms are not completely understood, the neurological effects of PPIs appear to be explained by the influence in ionic pumps controlling the membrane potential in neurons [38]. The lysosomes of patients taking PPIs seem to be less acidic than those of patients not taking them, which may make cells less able to degrade amyloid-beta protein, the principal substance that accumulates in the brain in patients with Alzheimer's disease [40-41]. Other hypotheses include that PPI and H2 receptor antagonist use have indirect effects related to systemic abnormalities (i.e., magnesium and vitamin B12 deficiency).

Cardiovascular disease

Over the past decade, PPI use has been associated with cardiovascular morbidity and mortality [42]. Increased risk of major acute cardiovascular events, including acute myocardial infarction and stroke, has been correlated with lengthy or high-dose treatment with PPIs [43-44]. Also, there is a concern that theoretical risk of malignant ventricular arrhythmias has been warned due to the development of hypomagnesemia, which may lengthen the QT interval and lead to torsade de pointes [45]. PPI use may lead to reduction of endothelial nitrous oxide levels through inhibition of dimethylarginine dimethylaminohydrolase enzymatic activity, which is responsible for clearance of asymmetric dimethylarginine, thereby reducing nitrous oxide synthase activity [46]. PPIs seem to increase the blood levels of chromogranin A, an important marker of neuroendocrine tumors that investigators have also proposed to be a biomarker of cardiovascular disease [47]. Chromogranin A and its derived peptides, vasostatins and catestatin, elicit vasodilatory and cardioregulatory effects that may be adaptive over the short term and maladaptive over the long term [48]. Finally, PPIs impair clopidogrel’s antiplatelet effect due to competition for the cytochrome P450 isoenzyme CYP2C19 [42].

Other adverse effects

Other, less prevalent side effects of PPIs are myopathy [49], hypomagnesemia [45], anemia, fundic gland polyps, micronutrient deficiencies, and subacute cutaneous lupus erythematosus [50].

## Conclusions

The risks and benefits of long-term PPI use should be carefully considered, especially in young patients, whose treatment with these drugs could last many years. Although there are multiple reported system-related side effects of these drugs, in most patients with appropriate short-term indications, the benefits of PPIs are likely to outweigh the risks.
